# Duration Dependent Outcomes of Combined Dorsal Root Ganglion Pulsed Radiofrequency and Epidural Steroid Injection in Chronic Lumbosacral Radicular Pain

**DOI:** 10.3390/jcm15020708

**Published:** 2026-01-15

**Authors:** Gülçin Babaoğlu, Nevcihan Şahutoğlu Bal, Ülkü Sabuncu, Şükriye Dadalı, Ali Çoştu, Şeref Çelik, Erkan Yavuz Akçaboy

**Affiliations:** Pain Clinic, Ankara Bilkent City Hospital, 06800 Ankara, Türkiye; nevci_sahut@hotmail.com (N.Ş.B.); sabuncuulku@gmail.com (Ü.S.); sukriyedadali@gmail.com (Ş.D.); alicostu@gmail.com (A.Ç.); drserefcelik@gmail.com (Ş.Ç.); akcaboyyavuz@gmail.com (E.Y.A.)

**Keywords:** lumbosacral radiculopathy, dorsal root ganglion, pulsed radiofrequency, chronic pain

## Abstract

**Background/Objectives:** The optimal duration of pulsed radiofrequency (PRF) applied to the dorsal root ganglion (DRG) remains unclear, particularly in patients with chronic lumbosacral radicular pain (LRP) who are unresponsive to conservative therapy. Although preclinical data suggest duration-dependent neuromodulatory effects, comparative clinical evidence for specific exposure times is limited. This study aimed to evaluate the outcomes of 4 min and 8 min DRG-targeted PRF applications performed in combination with transforaminal epidural steroid injection (TFESI) in patients with chronic LRP unresponsive to conservative treatment, to determine whether prolonged exposure provides superior analgesic and functional outcomes. **Methods:** In this prospective, single-center, observational comparative study, 72 patients with chronic lumbar radicular pain (LRP) refractory to conservative management received DRG-targeted PRF using standardized parameters (45 V, 20 ms, 2 Hz, ≤42 °C). Participants underwent either 4 min (*n* = 36) or 8 min (*n* = 36) PRF, assigned according to clinical discretion. All procedures were followed by transforaminal epidural injection of dexamethasone and bupivacaine. The primary endpoint was Numeric Rating Scale (NRS) pain intensity at 6 months. Secondary endpoints included Oswestry Disability Index (ODI), patient satisfaction, responder rates, and analgesic use across 1-, 3-, and 6-month follow-up. **Results:** Both groups achieved significant improvements from baseline at all time points. Linear mixed-effects analysis demonstrated a significant overall association favoring the 8 min protocol for pain (estimate: −0.81, 95% CI: −1.52 to −0.10, *p* = 0.025) and functional disability (estimate: −12.84, 95% CI: −19.36 to −6.32, *p* < 0.001). Functional benefits emerged by 3 months (*p* = 0.006), while pain reduction reached borderline statistical significance at 6 months (*p* = 0.048). The 8 min group showed numerically higher responder rates and patient satisfaction without increased adverse events. **Conclusions:** In this study evaluating a combined PRF and corticosteroid injection protocol, 8 min PRF exposure was associated with superior pain and functional outcomes compared to 4 min, without compromising safety. However, the observational design and concurrent medication administration limits causal inference. Randomized controlled trials are needed to confirm these findings and isolate the independent effect of PRF duration.

## 1. Introduction

Lumbosacral radicular pain (LRP) is a common neuropathic pain condition characterized by radiating pain along lumbar or sacral dermatomes, frequently extending below the knee. Its prevalence in the general population ranges from approximately 10% to 25%, making it one of the most frequent neuropathic pain syndromes. LRP typically manifests with both nociceptive and neuropathic features, and its chronic form is often associated with significant disability and reduced quality of life [1,2].

Management strategies for LRP include conservative modalities, pharmacologic treatments, and interventional procedures. Although physical therapy and exercise remain widely used conservative interventions, their effectiveness is variable [3]. Epidural steroid injections (ESIs) are frequently used, particularly in subacute phases, and may provide short-term analgesia, although their superiority to other therapies remains uncertain [4]. In chronic and refractory cases, pulsed radiofrequency (PRF) treatment of the dorsal root ganglion (DRG) has shown promise, as studies have indicated that it may lead to significant pain reduction and improved quality of life [5,6].

PRF is a neuromodulatory technique used in the management of chronic pain [7]. It delivers high-voltage currents in short bursts, maintaining tissue temperatures below the ablative levels, thereby providing analgesic effects without causing overt tissue damage. Its non-destructive nature makes it an attractive option compared to traditional continuous radiofrequency, which applies higher temperatures to the nerve tissues [8]. Proposed mechanisms include alterations in neuronal and glial activity, modulation of synaptic transmission, changes in inflammatory signaling, and effects on nociceptive gene expression [9]. The DRG represents a rational therapeutic target because it is an early site of pathophysiological change in neuropathic pain [10]. Despite widespread use, the results regarding the efficacy of PRF applied specifically to the lumbar DRG are heterogeneous, and it has been suggested that the duration of the effect may be shorter than in other regions [11,12]. This raises the question of how PRF settings (voltage, frequency, pulse width, and application duration) determine the clinical outcomes.

Preclinical models suggest that analgesic efficacy may increase with higher total electric field exposure. Clinically, applications exceeding 360 s may be associated with more pronounced pain improvement in the early period (e.g., 3 months) [13]. In contrast, protocols extended to 12 min may increase potential neural stress/damage without providing additional analgesic benefits [14]. Human studies comparing specific time points, such as 4 and 8 min of DRG-targeted PRF, are limited in the current literature [15,16]. Although preclinical findings and limited clinical data suggest that 6–8 min of exposure may have a stronger and more sustainable analgesic potential than 2–4 min, these inferences require confirmation via robust, prospective studies in the LRP population.

In routine practice, DRG-targeted PRF is frequently performed in combination with transforaminal epidural steroid injection (TFESI), reflecting both pragmatism and the potential for synergistic neuromodulatory and anti-inflammatory effects. However, this combined approach may obscure the independent contribution of PRF duration to clinical outcomes.

The present study aimed to evaluate the duration-dependent outcomes of DRG-targeted PRF in combination with TFESI by comparing 4 min and 8 min applications in patients with chronic LRP. We hypothesized that longer PRF exposure would be associated with superior analgesic and functional outcomes over short- and mid-term follow-up intervals. By standardizing all PRF parameters (voltage, frequency, pulse width) except duration, this study sought to determine an evidence-based optimal exposure time for improving outcomes in patients with difficult-to-treat chronic LRP.

## 2. Materials and Methods

### 2.1. Study Design

This single-center, prospective, non-randomized observational study was conducted at a tertiary pain clinic (Ankara Bilkent City Hospital, Ankara, Türkiye). This study was approved by the Institutional Ethics Committee (approval number: TABED 1-24-764; date: 4 December 2024) and was prospectively registered at ClinicalTrials.gov (NCT06748469) prior to patient enrollment. All participants provided written informed consent prior to enrollment. The study adhered to the principles of the Declaration of Helsinki and Good Clinical Practice guidelines. Between 23 December 2024 and 17 April 2025, consecutive patients with chronic LRP who met eligibility criteria were enrolled. PRF was performed as part of routine clinical practice, and the duration of PRF exposure (4 or 8 min) was determined by the treating physician based on individual clinical judgment. No systematic allocation scheme was used. The reporting of this study follows the Strengthening the Reporting of Observational Studies in Epidemiology (STROBE) guidelines.

### 2.2. Patient Population

#### 2.2.1. Inclusion Criteria

- Age ≥ 18 years.
- Chronic LRP persisting for ≥ 12 weeks.
- Insufficient pain relief (NRS ≥ 4) despite at least 4 weeks of conservative management, including physical therapy and/or pharmacological treatment (e.g., NSAIDs, gabapentinoids, or duloxetine).
- Radiological evidence of nerve root compression on lumbar magnetic resonance imaging (MRI), attributed to a herniated intervertebral disk (HIVD) or spinal stenosis.
- Persistent or recurrent radicular pain (NRS ≥ 4) at the time of PRF evaluation despite receiving at least one TFESI > 3 weeks prior.

#### 2.2.2. Exclusion Criteria

- Patient refusal.
- Lumbar radicular pain due to malignancy or infection.
- Diagnosis of diabetes mellitus and/or polyneuropathy.
- Diagnosis of demyelinating disorder (e.g., multiple sclerosis).
- Predominant axial low back pain.
- Neuropsychiatric conditions that would hinder follow-up and assessment (e.g., dementia and severe psychiatric disorders).
- Progressive motor weakness or cauda equina syndrome requiring urgent surgical intervention.
- Active systemic infection or infection at the injection site.
- Cardiac implantable electronic device.
- Known allergy to local anesthetic agents or contrast media.
- Presence of bleeding or coagulation disorders or ongoing use of oral anticoagulants.

### 2.3. Group Allocation

Patients were assigned to either the 4 min or 8 min PRF group according to clinical discretion at the time of the procedure. There was no randomization or pre-specified allocation rule, and the decision was made based on the treating physician’s preference.

### 2.4. Treatment Protocols

All procedures were performed under fluoroscopic guidance with patients in the prone position. After sterile preparation, local anesthesia was achieved using 2% prilocaine. A 22-gauge RF cannula with a 10 mm active tip (SMK™, Abbott Medical, Abbott Park, IL, USA) was advanced toward the target DRG. Correct needle placement was confirmed using sensory stimulation at 50 Hz with a threshold ≤0.4 V producing concordant paresthesia, motor stimulation at 2 Hz with a threshold ≥1.5 times the sensory threshold, and impedance values <500 Ω. The PRF parameters were standardized as follows: maximum output voltage, 45 V; pulse width, 20 ms; frequency, 2 Hz; and tip temperature ≤ 42 °C. In the 4 min group, PRF was applied for 240 s, and in the 8 min group, it was applied for 480 s. Following PRF, the cannula was slightly withdrawn, and epidural spread was confirmed using non-ionic contrast medium. Following PRF, the intervention was consistently combined with a transforaminal epidural steroid injection (TFESI) protocol. A 4 mL injectate containing dexamethasone (8 mg) and bupivacaine (5 mg) was delivered at each treated level. All adverse events during and after the procedure were documented.

### 2.5. Outcome Measures and Data Collection

Baseline assessments included demographic characteristics (age, sex, body mass index); comorbidities and use of anticoagulant/antithrombotic agents, pain characteristics such as pain localization (side, dermatome), total pain duration (months), and pain intensity (NRS 0–10); analgesic (nonsteroid anti-inflammatory drugs (NSAIDs), opioids) and adjuvant medication use (gabapentinoids, duloxetine, tricyclic antidepressants) history of physical therapy or lumbar surgery, and functional disability measured using the Turkish version of the ten-item Oswestry Disability Index (ODI) questionnaire (range: 0–100; 0 = no disability) [17]. In addition, procedural characteristics, such as the target level and target DRG, were documented.

Follow-up evaluations were performed by a blinded assessor at 1, 3, and 6 months. Pain intensity was evaluated using the NRS; analgesic use was assessed using a 4-point Likert scale (stopped/decreased/similar/increased); physical function was measured using the ten-item ODI questionnaire; and improvement was evaluated using the Global Perceived Effect of Satisfaction (GPES) using a 7-point Likert scale. Patient satisfaction was measured using a 5-point Likert scale (very satisfied to not satisfied at all). Clinically meaningful improvement was defined as a ≥50% reduction in NRS, a ≥30% or ≥10-point absolute reduction in ODI, and a GPES score of ≥6. Adverse events were assessed intra-procedurally, within the first 24 h, and at all subsequent follow-up visits.

### 2.6. Power Analysis

A priori power analysis was performed for the primary endpoint, defined as the between-group difference in NRS pain intensity at 6 months. The analysis was based on an ANCOVA framework, adjusting for baseline values. The minimum clinically important difference (MCID) for the primary endpoint was defined as 2.0 points on the NRS. Assuming a standard deviation of 2.5, a baseline–follow-up correlation of 0.50, a two-sided α of 0.05, and 90% power, a total sample size of 56 patients was required to detect this difference. A planned sample size of 72 patients (36 per group) was therefore deemed sufficient to detect differences within this range.

### 2.7. Statistical Analysis

All statistical analyses were performed using Python (version 3.14.0) within the Jupyter Notebook (version 7.5.1) environment. Data handling and descriptive statistics were conducted using the pandas and scipy.stats libraries. The primary longitudinal analyses, including linear mixed-effects (LME) and generalized estimating equations (GEE) models, were fitted using the statsmodels library.

Baseline characteristics were compared between the 4 min and 8 min groups using appropriate statistical analyses. Continuous variables were assessed for normality, and non-normally distributed variables are reported as median (IQR) and compared using the Mann–Whitney U test. Categorical variables are reported as frequencies (%) and were compared using the chi-square or Fisher’s exact test, as appropriate.

The longitudinal outcomes were changes in the NRS pain and ODI scores from baseline to 1, 3, and 6 months. Absolute and percentage changes were calculated for both outcomes. Between-group comparisons of changes at each time point were performed using the Mann–Whitney U-test. Responder rates were compared using chi-square tests, and differences were presented as percentages. Additional categorical outcomes included patient satisfaction (satisfied vs. not satisfied) and medication changes (decreased/stopped vs. unchanged/increased) were also assessed.

Although the power analysis was based on an ANCOVA framework to provide a conservative sample size estimate for the primary endpoint at a single time point, linear mixed-effects (LME) models were the primary approach used to evaluate treatment effects over time, while accounting for repeated measures within subjects. These longitudinal models were preferred as they more efficiently utilize all repeated measures across the 6-month follow-up period and are more robust to missing data than standard ANCOVA, thereby enhancing the overall statistical power and reliability of the study results. The models included fixed effects for group (8 min vs. 4 min), time (categorical), and their interaction, with random intercepts for the subjects. We report the overall group effect and month-specific contrasts with 95% confidence intervals; negative estimates indicate the superiority of the 8 min protocol. The model assumptions were verified by examining the distribution of residuals. As the residuals for the ODI score model demonstrated a non-normal distribution, a sensitivity analysis using generalized estimating equations (GEE) with an exchangeable working correlation was fitted to obtain population-averaged estimates that were robust to correlation structure misspecification. All tests were two-sided with α = 0.05. Month-specific *p*-values from the LME and GEE models were not adjusted for multiple comparisons and should be regarded as exploratory.

Participants with at least one post-intervention follow-up measurement were included in LME and GEE models, allowing individuals with incomplete follow-up to contribute data from observed time points. For time-point–specific comparisons, analyses were performed using available data at each respective follow-up visit, resulting in varying sample sizes across time points, as reflected in the tables. No imputation methods were applied.

## 3. Results

**Figure 1:**
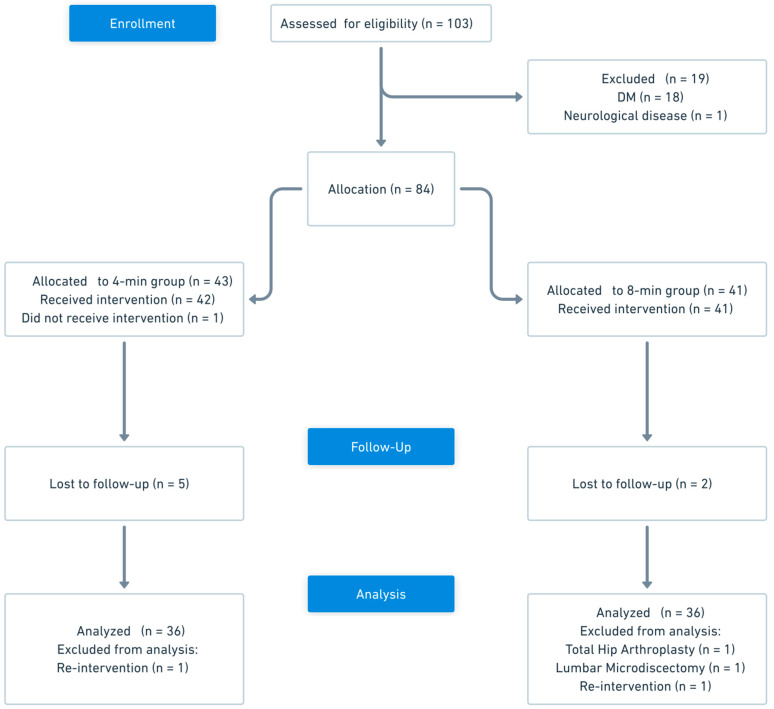
Flow chart of study.

Between 23 December 2024 and 17 April 2025, a total of 103 patients were assessed for eligibility. Of these, 19 patients were excluded prior to allocation: 18 patients with diabetes mellitus and 1 patient with multiple sclerosis. After exclusions, 84 patients were deemed eligible for this study. These individuals were allocated to two groups according to clinicians’ procedural preference: 43 patients to the 4 min group and 41 patients to the 8 min group (total *n* = 84 allocated). A total of 72 patients (*n* = 36 per group) contributed post-intervention follow-up data and were included in the final longitudinal analyses (Figure 1).

Baseline demographic and clinical characteristics were well balanced between the two treatment groups (Table 1). The median age was 58.5 years in the 4 min group and 53.5 years in the 8 min group (*p* = 0.073). Body mass index, baseline pain intensity (NRS), and functional disability (ODI) did not differ significantly between the groups. Baseline median NRS pain scores were 8.0 in both groups, while median ODI scores were 55.0 and 54.0 in the 4 min and 8 min groups, respectively. The median pain duration was 12.0 months in both groups. No significant differences were observed in sex distribution, medication use, physical therapy participation, or prior surgical intervention between the two groups (Table 1). The most frequently targeted DRG levels were L4-5/L5-S1 (55.6% of all patients), followed by L5-S1/S1 (18.1%) and L3-4/L4-5 (11.1%). The distribution of the targeted levels was similar between the 4 min and 8 min groups.

Both treatment groups demonstrated substantial improvements in pain intensity and functional disability scores at all the follow-up time points. The 8 min group showed consistently greater improvements than the 4 min group, although the differences in median changes did not reach statistical significance in the univariate analyses (Table 2).

**Figure 2:**
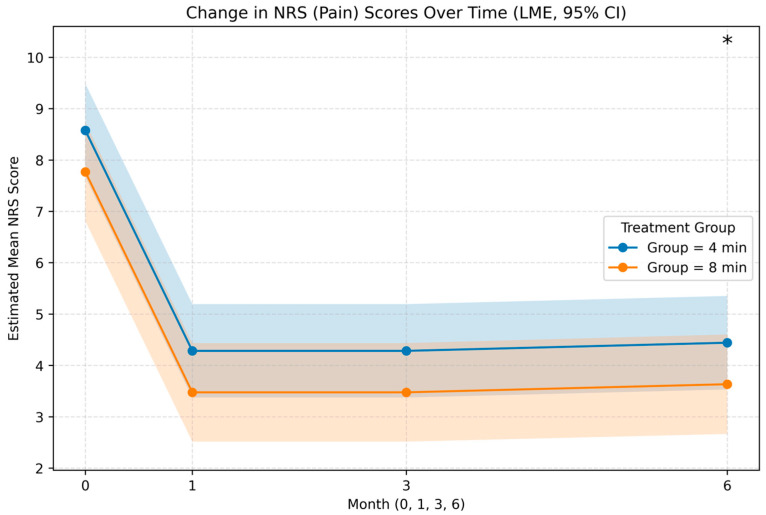
Longitudinal changes in Numeric Rating Scale (NRS) pain scores over 1, 3, and 6 months. Both groups improved from baseline, with numerically greater reductions observed in the 8 min protocol across follow-up; between-group differences reached borderline statistical significance only at 6 months. * Statistically Significant.

At 1 month, both groups achieved a median absolute NRS reduction of 4.0 points, representing a median percent reduction of 50.0% in both groups. By 6 months, the 8 min group maintained a median absolute reduction of 5.0 points compared to 3.0 points in the 4 min group, although this difference was not statistically significant (*p* = 0.215) (Figure 2).

**Figure 3:**
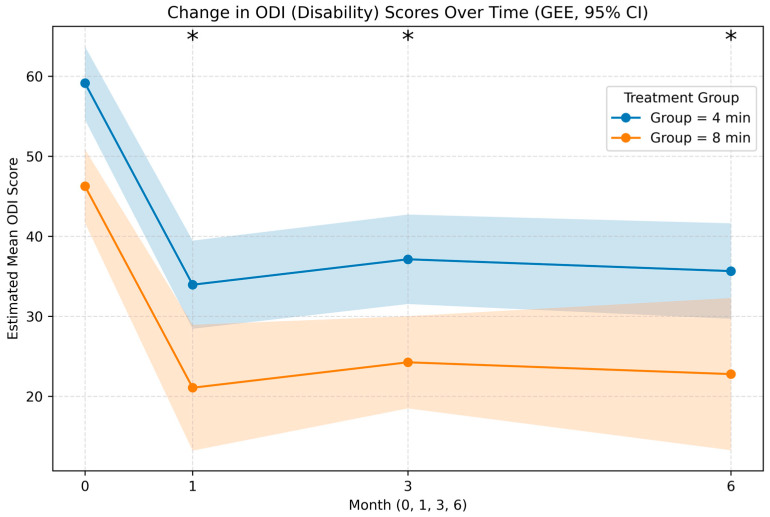
Longitudinal changes in Oswestry Disability Index (ODI) scores over 1, 3, and 6 months. Functional improvement was observed in both groups, with statistically significant and consistently greater reductions in disability associated with the 8 min protocol, particularly at 3 and 6 months. * Statistically Significant.

For functional disability, the 8 min group demonstrated greater median improvement in the ODI scores at all time points. At 6 months, the median absolute ODI reduction was 28.0 points in the 8 min group versus 17.0 points in the 4 min group (*p* = 0.153), corresponding to median percent improvements of 57.1% and 33.5%, respectively (*p* = 0.104) (Figure 3).

At 1 month, the responder rates were similar between the groups for all outcome measures. The NRS responder rates were identical in both groups. By 6 months, the 8 min group showed numerically higher responder rates, with 62.9% achieving ≥ 50% NRS reduction compared to 50.0% in the 4 min group (*p* = 0.270). ODI responder rates were consistently higher in the 8 min group, reaching 74.3% at 6 months compared with 66.7% in the 4 min group (*p* = 0.480). GPES responder rates (score ≥ 6) showed a trend favoring the 8 min protocol at later time points, with 57.1% of patients in the 8 min group at 6 months versus 36.1% in the 4 min group (*p* = 0.082), representing a 21.0 percentage point difference. Patient satisfaction and medication reduction rates were numerically higher in the 8 min group at all follow-up time points, although the differences were not statistically significant (Table 3).

Linear mixed-effects modeling revealed a statistically significant overall association favoring the 8 min protocol for pain reduction (NRS estimate: −0.81, 95% CI: −1.52 to −0.10, *p* = 0.025). When examining time-specific differences, the between-group difference in NRS reached borderline statistical significance only at 6 months (estimate: −1.35, 95% CI: −2.70 to −0.01, *p* = 0.048) (Table 4). For functional disability outcomes, the 8 min protocol demonstrated a highly significant overall advantage (LME estimate: −12.84, 95% CI: −19.36 to −6.32, *p* < 0.001). This finding was confirmed using generalized estimating equations as a robust alternative method (GEE estimate: −12.87, 95% CI: −18.76 to −6.98, *p* < 0.001). Month-specific differences favored the 8 min Group at 3 months (LME estimate: −17.79, *p* = 0.006; GEE estimate: −17.81, *p* = 0.001) and persisted at 6 months (LME estimate: −17.23, *p* = 0.009; GEE estimate: −17.27, *p* = 0.002) (Table 4).

Adverse events were systematically monitored throughout the study period and are summarized. One patient experienced an accidental dural puncture during the procedure; however, no post-dural puncture headache occurred. Except for mild discomfort during needle placement and localized ecchymosis at the puncture site, no significant adverse events were observed. Overall, both PRF protocols were well tolerated with no serious adverse events reported in either group.

## 4. Discussion

This prospective comparative study found that both 4- and 8 min DRG-targeted PRF, performed in combination with TFESI, were associated with significant improvements in pain intensity and functional outcomes in patients with chronic LRP. The 8 min protocol was associated with greater clinical improvement without increasing adverse events. Longitudinal mixed-effects analyses supported an overall association favoring longer PRF exposure with functional improvement emerging earlier and more prominently in the 8 min group, while pain reduction reached borderline statistical significance at six months. Mid- and long-term follow-up findings also showed similar trends in global improvement, patient satisfaction and reduced analgesic use. These results suggest that PRF duration is a potentially critical and modifiable factor associated with greater clinical improvement and that rational optimization of exposure time may enhance neuromodulatory outcomes without compromising safety.

Unlike conventional thermal radiofrequency ablation, PRF achieves analgesia through neuromodulation rather than heat-induced tissue destruction and is therefore generally regarded as a non- or minimally neurodestructive modality [7,8,18]. Proposed mechanisms include microscopic and biochemical alterations in neural and glial cells, modulation of synaptic transmission and perineural microarchitecture, and regulation of inflammatory responses and nociceptive gene expression [9,19]. Clinically, consistent PRF-specific adverse effects have not been reported, and experimental observations of delayed dorsal horn activity following DRG exposure support a nonthermal mechanism [20,21]. Nevertheless, PRF may not be entirely non-destructive, as ultrastructural damage to mitochondria, microfilaments, and microtubules, which are more prominent in C fibers, has been observed after PRF exposure [22]. Moreover, extending the exposure duration does not always enhance antiallodynic effects; in fact, increased ATF3 mRNA expression levels have been associated with greater neuronal stress and injury [14].

Experimental and clinical evidence collectively indicate that the relationship between PRF exposure time and analgesic efficacy is non-linear; that is, extending the duration enhances therapeutic effects up to an intermediate threshold, beyond which further prolongation may yield diminishing or even adverse returns. In preclinical models such as chronic constriction injury (CCI) and spinal nerve ligation (SNL), exposure durations between 6- and 8 min produced stronger and more sustained antinociceptive responses than shorter applications while maintaining histological safety [13,14,23]. For instance, an 8 min PRF application improved resting paw posture scores and reduced IL-6 expression without increasing tissue damage, whereas a 6 min exposure achieved significant and durable antiallodynic effects [13]. These findings suggest that short applications may insufficiently engage neuromodulatory mechanisms, whereas moderate extensions (6–8 min) more effectively modulate nociceptive signaling and inflammatory pathways.

Our findings indicate that both 4 min and 8 min PRF protocols combined with TFESI were associated with greater improvements in pain and function, with consistently greater benefits observed in the 8 min group across all time points. The superiority of the longer exposure was most pronounced for functional disability, reflected by approximately 13-point lower ODI scores (*p* < 0.001), with differences emerging by the first month and becoming statistically robust thereafter, while pain reduction achieved significance at six months. These temporal dynamics suggest that PRF duration may differentially modulate neuroinflammatory and nociceptive mechanisms through cumulative neuromodulatory effects. Importantly, the extended 8 min exposure did not compromise safety.

The broader clinical literature provides additional support for a duration-dependent but non-linear efficacy relationship in PRF treatment of radicular pain. A meta-analysis comparing lumbar epidural steroid injections with DRG-PRF showed that total PRF durations exceeding 360 s yielded significantly greater pain reduction at three months, whereas shorter protocols (≤360 s) failed to demonstrate this advantage [24]. Similarly, randomized controlled trials have shown that intermediate durations outperform both shorter (2–4 min) and excessively prolonged (≥12 min) applications [15,25]. One trial comparing 4-, 6-, and 8 min regimens, all protocols improved pain and function, but the 6 min duration produced the most consistent and statistically robust benefits, while the 8 min application provided incremental gains, particularly in patient satisfaction (GPE) and functional recovery [15]. In contrast, a study comparing 6- and 12 min protocols reported no additional analgesic benefit with prolonged stimulation, supporting a saturation threshold beyond which further exposure fails to augment neuromodulation and may risk subclinical neuronal stress [25].

Our findings align with current experimental and clinical evidence indicating that PRF duration is a key determinant of therapeutic efficacy. The superior outcomes observed with the 8 min protocol compared with the 4 min exposure, when applied in combination with TFESI, support the view that moderately extended durations enhance neuromodulatory engagement and overall therapeutic benefit. Improvements observed at 3 and 6 months suggest that longer stimulation within this safe exposure range contributes to more durable pain relief and functional recovery without compromising safety. The absence of procedure-related complications further supports the safety of the applied parameters (45 V, 20 ms pulse width, 2 Hz frequency, ≤42 °C).

Taken together, the growing body of preclinical and clinical evidence suggests that the relationship between PRF duration and treatment efficacy is non-linear—therapeutic benefits increase up to an optimal range of approximately 6–8 min, beyond which gains plateau. From a clinical standpoint, PRF duration should therefore be considered a modifiable procedural parameter rather than an arbitrary setting. For DRG-targeted PRF in chronic lumbosacral radicular pain, a standardized exposure of 6–8 min appears to offer the most favorable balance between efficacy and safety.

This study has several limitations that should be considered when interpreting the findings. The most important is the non-randomized allocation of patients based on physician preference. Treating physicians may have preferentially selected longer PRF durations for patients with unmeasured clinical characteristics, which could independently influence outcomes and may restrict causal inference. Although baseline variables were comparable, residual confounding cannot be excluded. The concomitant use of dexamethasone and bupivacaine following PRF precludes isolation of the independent effect of PRF duration, as the observed improvements likely reflect a combined neuromodulatory and anti-inflammatory response. The sample size (*n* = 72), while close to the powered target, limited statistical precision for detecting smaller yet potentially meaningful differences and prevented subgroup analyses. Loss to follow-up after the intervention (7/83 patients) may have introduced attrition bias, as these individuals did not return for post-procedural follow-up assessments. Furthermore, the single-center design and strict inclusion criteria limit the generalizability of the results beyond a tertiary referral population and are a barrier to external validity. Finally, the 6-month follow-up period captures only intermediate outcomes; longer observation is needed to evaluate the durability of the benefit and detect delayed complications. Despite these constraints, the prospective design, procedural standardization, and consistent analytic approach strengthen confidence that the observed associations favor 8 min combined protocol, underscoring PRF duration as a key modifiable parameter deserving further validation in randomized controlled trials.

## 5. Conclusions

This prospective comparative study found that extending PRF exposure from 4 to 8 min at the dorsal root ganglion enhanced both analgesic and functional outcomes in patients with chronic lumbosacral radicular pain without increasing the adverse effects. The 8 min protocol provided a statistically significant improvement in pain and disability, suggesting that treatment duration plays a key role in optimizing neuromodulatory outcomes. However, definitive conclusions are constrained by the study’s non-randomized allocation and the concurrent corticosteroid administration, which prevents isolation of the independent PRF effect. When integrated with experimental and clinical evidence from the literature, these results are consistent with a non-linear, duration-dependent relationship in which the therapeutic effect increases up to an intermediate range of approximately 6–8 min but plateaus or potentially reverses with longer exposures. Our findings suggest that PRF duration represents a clinically relevant and potentially modifiable procedural parameter and an exposure window of 6–8 min appears to offer the most favorable balance between efficacy and safety for DRG-PRF therapy in patients with chronic lumbosacral radicular pain.

## Figures and Tables

**Table 1:** Baseline demographic and clinical characteristics of study participants.

	4 min (*n* = 36)	8 min (*n* = 36)	*p* Value
Age, median (IQR) (years)	58.5 (54.0–68.5)	53.5 (41.0–63.2)	0.073
BMI, median (IQR)	27.0 (24.4–31.1)	25.9 (24.4–28.6)	0.714
NRS baseline, median (IQR)	8.0 (7.0–9.0)	8.0 (7.8–9.0)	0.718
ODI baseline, median (IQR)	55.0 (43.5–64.0)	54.0 (41.5–70.5)	0.685
Pain duration (months), median (IQR)	12.0 (6.0–39.0)	12.0 (6.0–24.0)	0.225
Sex, female (%)	58.3%	63.9%	0.629
NSAID (%)	97.2%	97.2%	1.000
Opioid (%)	22.2%	13.9%	0.358
Gabapentinoid (%)	13.9%	8.3%	0.453
Duloxetine (%)	22.2%	13.9%	0.358
Physical therapy (%)	80.6%	77.8%	0.772
Surgery (%)	13.9%	19.4%	0.527

Abbreviations: *n* = number of patients; BMI = body mass index; NRS = Numeric Rating Scale; ODI = Oswestry Disability Index; IQR = interquartile range; NSAID = nonsteroidal anti-inflammatory drug(s).

**Table 2:** Temporal changes in pain scores and functional disability index.

Outcome	Time	4 min (*n* = 36)	8 min (*n* = 36)	*p* Value
NRS—Absolute change	1 mo	−4.0 (−5.0, −2.0)	−4.0 (−6.0, −2.8)	0.283
NRS—Percent change (%)	1 mo	−50.0 (−57.9, −25.0)	−50.0 (−75.7, −33.3)	0.238
ODI—Absolute change	1 mo	−22.0 (−32.0, −4.0)	−24.0 (−40.0, −10.0)	0.457
ODI—Percent change (%)	1 mo	−43.9 (−58.1, −7.2)	−46.1 (−76.5, −20.9)	0.478
NRS—Absolute change	3 mo	−3.0 (−5.0, −2.0)	−4.5 (−6.0, −3.0)	0.220
NRS—Percent change (%)	3 mo	−45.0 (−64.7, −25.0)	−56.3 (−75.7, −37.5)	0.200
ODI—Absolute change	3 mo	−21.0 (−32.5, −5.5)	−27.0 (−46.5, −10.0)	0.138
ODI—Percent change (%)	3 mo	−37.2 (−64.4, −7.9)	−54.2 (−78.3, −23.2)	0.093
NRS *—Absolute change	6 mo	−3.0 (−6.0, −1.0)	−5.0 (−6.5, −2.0)	0.215
NRS *—Percent change (%)	6 mo	−43.8 (−75.0, −19.2)	−62.5 (−82.9, −35.4)	0.141
ODI *—Absolute change	6 mo	−17.0 (−38.0, −3.5)	−28.0 (−46.0, −9.0)	0.153
ODI *—Percent change (%)	6 mo	−33.5 (−64.1, −6.4)	−57.1 (−85.3, −19.1)	0.104

Abbreviations: mo: month; *n* = number of patients; NRS = Numeric Rating Scale; ODI = Oswestry Disability Index; IQR = interquartile range. * 1 missing patient.

**Table 3:** Proportion of treatment responders and patient satisfaction metrics.

Outcome	Month	4 min	8 min	χ^2^ (1)	*p* Value
GPES responder (≥6)	1	19/36 (52.8%)	18/36 (50.0%)	0.06	0.812
Patient satisfaction (satisfied)	1	26/36 (72.2%)	25/36 (69.4%)	0.07	0.802
Medication decreased/stopped	1	19/36 (52.8%)	21/36 (58.3%)	0.22	0.642
GPES responder (≥6)	3	17/36 (47.2%)	24/36 (66.7%)	2.78	0.102
Patient satisfaction (satisfied)	3	22/36 (61.1%)	24/36 (66.7%)	0.24	0.622
Medication decreased/stopped	3	17/36 (47.2%)	23/36 (63.9%)	2.02	0.152
GPES responder (≥6)	6	13/36 (36.1%)	20/35 (57.1%)	3.16	0.082
Patient satisfaction (satisfied)	6	18/36 (50.0%)	23/35 (65.7%)	1.80	0.182
Medication decreased/stopped	6	17/36 (47.2%)	21/35 (60.0%)	1.16	0.282
NRS responder (≥50%)	1	20/36 (55.6%)	20/36 (55.6%)	0.00	1.000
NRS responder (≥50%)	3	18/36 (50.0%)	23/36 (63.9%)	1.42	0.230
NRS responder (≥50%)	6	18/36 (50.0%)	22/35 (62.9%)	1.19	0.270
ODI responder (≥30%)	1	25/36 (69.4%)	28/36 (77.8%)	0.64	0.420
ODI responder (≥30%)	3	24/36 (66.7%)	29/36 (80.6%)	1.79	0.180
ODI responder (≥30%)	6	24/36 (66.7%)	26/35 (74.3%)	0.49	0.480

Abbreviations: GPES = Global perceived effect of satisfaction; NRS = Numeric Rating Scale; ODI = Oswestry Disability Index.

**Table 4:** Between-group differences in NRS and ODI from linear mixed-effects models (negative estimates favor 8 min).

NRS	Month	Estimate	Std. Error (SE)	95% CI (Lower, Upper)	*p* Value
**Main Effect**	Overall	−0.81	0.36	−1.52, −0.10	0.025
**Month-Specific Differences**	0	0.41	0.67	−0.91, 1.72	0.544
	1	−1.21	0.67	−2.53, 0.10	0.071
	3	−1.09	0.67	−2.41, 0.22	0.103
	6	−1.35	0.69	−2.70, −0.01	0.048
**ODI**	**Month**	**Estimate**	**Std. Error (SE)**	**95% CI (Lower, Upper)**	***p*** **Value**
**Main effect**					
LME (Primary model)	Overall	−12.84	3.33	−19.36, −6.32	<0.001
GEE (Robust alternative)	Overall	−12.87	3.01	−18.76, −6.98	<0.001
**Month-Specific Differences**					
LME (Primary model)	0	−4.67	6.43	−17.27, 7.94	0.468
	1	−11.87	6.43	−24.47, 0.74	0.065
	3	−17.79	6.43	−30.40, −5.18	0.006
	6	−17.23	6.56	−30.10, −4.37	0.009
GEE (Robust alternative)	0	−4.69	2.93	−10.44, 1.06	0.110
	1	−11.89	3.13	−18.03, −5.75	<0.001
	3	−17.81	5.44	−28.47, −7.15	0.001
	6	−17.27	5.58	−28.20, −6.33	0.002

Abbreviations: NRS = Numeric Rating Scale; ODI = Oswestry Disability Index.

## Data Availability

The raw data supporting the conclusions of this article will be made available by the corresponding author, G.B., upon reasonable request.

## Abbreviations

The following abbreviations are used in this manuscript:

BMI	Body mass index
CCI	Chronic constriction injury
CI	Confidence interval
DM	Diabetes mellitus
DRG	Dorsal root ganglion
ESI	Epidural steroid injection
GEE	Generalized estimating equations
GPES	Global Perceived Effect of Satisfaction
HIVD	Herniated intervertebral disk
IQR	Interquartile range
LME	Linear mixed-effects
LRP	Lumbosacral radicular pain
MRI	Magnetic resonance imaging
*n*	Number of patients
NRS	Numeric Rating Scale
NSAID	Nonsteroidal anti-inflammatory drug
ODI	Oswestry Disability Index
PRF	Pulsed radiofrequency
SE	Standard Error
SNL	Spinal nerve ligation
TFESI	Transforaminal epidural steroid injection
